# EEG assessment of artificial intelligence-generated content impact on student creative performance and neurophysiological states in product design

**DOI:** 10.3389/fpsyg.2025.1508383

**Published:** 2025-07-02

**Authors:** Shuxin Wang, Xin Tao, Hongbo Ma, Fanglian Li, Chuanqi Wu

**Affiliations:** ^1^Changzhou Vocational Institute of Textile and Garment, College of Creative Design, Changzhou, China; ^2^Chizhou University, College of Arts and Education, Chizhou, China

**Keywords:** creative performance, product design, artificial intelligence, electroencephalography, concentration level, relaxation level

## Abstract

**Objectives:**

The purpose of this study has been to evaluate the use of Artificial Intelligence-Generated Content (AIGC) tools in design education, in terms of their effects on creative performance, concentration, and relaxation levels, for university students enrolled in an undergraduate design program.

**Methods:**

An experimental design was implemented, using two groups differentiated by their design tool usage (AIGC tools versus traditional software). The sample consisted of 64 third-year undergraduate design students from a public university in Eastern China. Participants completed a three-hour intelligent walking cane design task. The AIGC group used ChatGPT, Midjourney, and Stable Diffusion, while the control group used traditional design software. Neurophysiological states were continuously monitored using BrainLink Pro EEG headband devices. Creative performance was evaluated using standardized design assessment criteria; concentration and relaxation levels were measured through EEG data analysis.

**Results:**

The study’s participants demonstrated that use of AIGC tools significantly enhanced creative performance (*M* = 115.13, SD = 6.44) compared to traditional methods (*M* = 110.69, SD = 9.37), *t*(62) = 2.208, *p* = 0.031, *d* = 0.55. The AIGC group showed significantly higher concentration levels (*M* = 51.06, SD = 2.54) than controls (*M* = 48.31, SD = 2.87), *t*(62) = 4.062, *p* < 0.001, *d* = 1.02. No significant difference was found in relaxation levels between groups (*p* = 0.191). Correlation analysis revealed a strong positive relationship between concentration level and creative performance (*r* = 0.67), while relaxation showed weaker associations (*r* = 0.29).

**Conclusion:**

This study has demonstrated that use of AIGC tools improves creative performance and concentration in design students, with the enhancement primarily driven by improved attentional focus and cognitive resource optimization. The integration of AIGC and EEG technologies provides objective neurophysiological evidence for understanding AI-assisted creativity in design education. It is suggested that AIGC tools should be incorporated into design curricula to enhance student creative outcomes while maintaining appropriate balance with traditional design methods.

## 1 Introduction

The application of Artificial Intelligence (AI) technology in the field of creative design is increasingly widespread (Cetinic and She, 2022). Artificial Intelligence-Generated Content (AIGC) (AIGC) technology, based on machine learning, has attracted significant attention due to its ability to automatically generate diverse design materials and ideas (Hitsuwari et al., 2023). Several studies have demonstrated that AIGC can inspire students, broaden their thinking, and enhance design efficiency (Hwang and Wu, 2025; Lee et al., 2024; Liu et al., 2024). However, scholars have also cautioned that excessive reliance on AI-generated content may inhibit students’ independent creative thinking, potentially leading to homogenization in design solutions (Magni et al., 2024). Furthermore, the quality and originality of AIGC have been subject to scrutiny, and the scope of its applicability in creative design remains to be fully delineated (Park et al., 2023).

While some studies, such as Grassini and Koivisto (2024), have compared the creative output generated by AIGC and traditional tools, these investigations have primarily relied on subjective assessments and behavioral performance indicators, with little exploration of students’ neurophysiological states and physiological responses during the design process. Design activities encompass not only creative thinking and problem-solving but are also intricately linked to factors such as concentration levels, relaxation levels, and affective experiences (Nguyen et al., 2018). As a novel design assistance tool, AIGC’s unique human-computer interaction modality may significantly influence students’ cognitive processes, brain states, and subjective experiences, consequently affecting both the process and outcomes of creative design (Hanafy, 2023).

To thoroughly investigate the impact of AIGC assistance on the design process and creative performance, employing objective measurement methods to detect students’ cognitive and affective states in real-time can provide a substantial complement to traditional subjective reports and behavioral analysis (Deng and Wang, 2019; Lai et al., 2019). Neurophysiological measurement techniques, such as Electroencephalography (EEG), with their high temporal resolution and sensitivity to cognitive processing, offer a potential solution to this challenge (Chen et al., 2017). By analyzing students’ EEG patterns under different conditions, researchers can reveal the impact of design processes on students’ concentration levels and relaxation states, and explore the relationship between these factors and creative design performance (Jia and Zeng, 2021). This not only helps in understanding the mechanism of AIGC-assisted design but also provides new insights for optimizing AI-enhanced creative design human-computer interaction in educational settings.

AIGC and EEG technologies offer novel paradigms and methods for creativity research. AIGC, by automatically generating diverse design concepts and visual solutions, provides individuals with inspiration, material support, and instant feedback, potentially expanding their creative thinking (Lee, 2022). EEG technology offers a novel perspective for creativity research through real-time neural activity monitoring, enabling researchers to identify neurophysiological markers of creative cognition and develop evidence-based interventions to enhance creative performance. While several studies have examined AI-assisted design and creative cognition (Anantrasirichai and Bull, 2022; Magni et al., 2024), the specific integration of AIGC tools with EEG measurements during product design tasks remains limited. This study extends this emerging literature by examining not only creative outcomes but also the real-time neurophysiological correlates of concentration and relaxation during the design process, offering insights into how AIGC tools impact neurophysiological states during creative work.

This study aims to advance the emerging field of combining AIGC technology with EEG technology. Specifically, it utilizes AIGC technology to assist students in generating inspiration and diverse creative content, while employing EEG technology to record and analyze their brain activity as they engage with the generated material. This exploration is expected to provide an empirical foundation for developing more effective AI creative support tools and educational strategies. Based on these considerations, this study proposes the following research questions (RQs):

*RQ1*: What are the differences in the effects of AI-assisted design tools and traditional design tools on college students’ creative performance?

*RQ2*: What are the differences in the effects of AI-assisted design tools and traditional design tools on college students’ neurophysiological states (concentration level and relaxation level)?

*RQ3*: What is the relationship between college students’ neurophysiological states (concentration level and relaxation level) and their creative performance? Does this relationship differ between AIGC-assisted design and traditional design conditions?

## 2 Literature review

### 2.1 Neurophysiological states in creative performance

Creative performance refers to the ability to produce novel and valuable ideas or works (Runco and Jaeger, 2012). Novelty implies originality, distinguishing creative works from copies or plagiarism. Value indicates that creative outputs should contribute meaningfully to society or solve practical problems in specific contexts, emphasizing both universal significance and context-specific relevance. Researchers have recognized the influence of multidimensional factors on creative performance, with concentration and relaxation levels playing particularly significant roles.

Concentration level refers to an individual’s ability to maintain and control cognitive resources on a specific task, including selective attention, sustained attention, and attention switching (Krauzlis et al., 2023). During the creative process, an individual’s attention state fluctuates between high concentration and relaxed thinking (Carruthers et al., 2018). Relaxation level is characterized by a state of low arousal and low stress, typically accompanied by physiological changes such as reduced muscle tension and slowed heart rate (Kora et al., 2021). In certain stages of design thinking, particularly when making distant associations, a relatively relaxed brain state can facilitate the generation of creative ideas by helping break conventional thinking patterns (Liu et al., 2021). Research by Yang et al. (2019) suggests that in complex problem-solving, brief distractions or relaxation may lead to an “unconscious thought effect,” potentially yielding more creative solutions.

The emergence of AIGC has sparked new considerations about the synergy between humans and artificial intelligence in the creative process. In an AIGC-assisted design environment, individuals’ neurophysiological processes and brain states, particularly those related to concentration and relaxation, may undergo changes. Understanding how these changes impact creative performance presents a critical area for further exploration.

### 2.2 AIGC applications in design

Artificial Intelligence-Generated Content (AIGC) technology employs machine learning algorithms to automatically generate creative content such as text, images, and audio (Liu et al., 2024). The advantages of AIGC technology are primarily reflected in its efficiency, diversity, and interactivity. It can rapidly generate a large volume of creative content, transcend the limitations of human thinking patterns to produce unanticipated innovative concepts, and support human-machine collaborative ideation. Consequently, these capabilities allow for personalized creative customization and iterative refinement of ideas, substantially improving the scale and speed of innovation across various domains (Chen and Chang, 2024).

In product design specifically, AIGC has transformed traditional design processes through several breakthrough applications (Huang et al., 2024). Regenwetter et al. (2022) demonstrated how generative adversarial networks (GANs) integrated with parametric modeling significantly reduced automotive design bikes from weeks to days, while simultaneously expanding design variation possibilities. Their research showed that designers using AIGC tools reported significantly higher satisfaction with final solutions compared to traditional methods. Building on this foundation, Li et al. (2025) investigated human-machine collaboration paths in AIGC-enabled product styling design, finding that the integration of generative AI technology with traditional design workflows resulted in enhanced efficiency and effectiveness in the product development process. This collaborative approach leveraged DALL-E’s visual generation capabilities combined with specialized constraint-satisfaction algorithms to ensure manufacturability of the proposed designs.

Various AI generation programs leveraging semantic analysis, deep learning, and intelligent algorithms are currently available in the market. Modern design-focused AIGC tools have evolved beyond simple text-to-image systems to incorporate domain-specific requirements. For instance, systems like Autodesk’s Dreamcatcher generate mechanical designs that satisfy specific physical constraints, while Midjourney and Stable Diffusion enable rapid visualization of complex product concepts from simple text prompts (Floridi and Chiriatti, 2020).

AIGC technology still faces limitations in content quality, creativity level, and evaluation feedback. The generated content often lacks human common sense knowledge and esthetic judgment, necessitating manual secondary screening and optimization (Sarker et al., 2024). These limitations directly impact the cognitive processes involved in design, as designers must continuously evaluate and refine AI-generated solutions, potentially altering their concentration patterns and mental states during creative work. This cognitive interaction between human designers and AIGC tools creates a unique opportunity for neurophysiological exploration through EEG technology. By monitoring brain activity during AIGC-assisted design processes, researchers can identify specific neural signatures associated with creative evaluation, decision-making, and ideation enhancement when working with AI tools—providing objective measures of how these technologies alter the fundamental cognitive aspects of design thinking beyond what subjective reports alone can reveal (Jiang et al., 2024). This neurophysiological perspective offers crucial insights for optimizing human-AI creative partnerships and understanding the cognitive mechanisms through which AIGC influences design performance.

### 2.3 EEG assessment of creative cognitive processes

Electroencephalography (EEG) is a neurophysiological method used to monitor and record brain electrical activity, serving as a tool to visualize cognitive processes (Niso et al., 2023; Yu et al., 2021). Compared to other brain imaging technologies, EEG-based devices offer advantages such as high temporal resolution, non-invasiveness, portability, and relatively low cost, making them popular in interactive learning research (Wu et al., 2024; Wu and Wang, 2024). Modern EEG devices, such as Emotiv and Neurosky, integrate electrodes, wireless transmission, and data processing modules, simplifying signal acquisition and real-time analysis. These devices allow users to view their EEG spectra and cognitive indicators like concentration and relaxation levels in real time through mobile apps or computer software (Dadebayev et al., 2022). Beyond scientific and medical applications, EEG technology is gradually entering mass-market scenarios such as electronic games, driving safety, and stress management (He et al., 2023). Current market EEG devices can identify different neurophysiological states with high accuracy (Reddy et al., 2022).

EEG signals can be categorized into five types of brain waves: *δ* (0.5–4 Hz, associated with deep sleep), *θ* (4–8 Hz, related to meditation and imagination), α (8–15 Hz, indicating a relaxed, awake state), β (15–32 Hz, linked to focus and concentration), and γ (associated with advanced mental activities) (Jebelli et al., 2018). In interactive learning environments, EEG technology is widely used to monitor individuals’ neurophysiological states in real-time, particularly focusing on relaxation (associated with α waves) and concentration levels (related to β and γ waves enhancement) (Davidesco et al., 2021). This information facilitates the development of adaptive learning materials or feedback strategies to optimize individuals’ neurophysiological states.

Recent studies have revealed intriguing connections between EEG indicators and creative thinking processes. The enhancement of posterior α waves is associated with distant association and divergent thinking in creative problem-solving, potentially reflecting a degree of relaxation (Huang and Chang, 2023). Frontal *θ* wave enhancement reflects attention concentration and working memory processing in creative thinking. While β and γ wave enhancement typically indicates increased attention levels, α wave suppression has been observed in some creative tasks and flow experiences, suggesting a complex relationship between relaxation and concentration in creative activities. This complexity underscores the importance of using EEG to simultaneously monitor relaxation and concentration levels, as their dynamic balance is crucial for understanding and promoting creative thinking in interactive learning environments.

The integration of EEG technology with AIGC-assisted design processes offers a methodological framework that allows for real-time assessment of neurophysiological states during creative activities. This approach enables researchers to move beyond retrospective self-reports or behavioral observations to directly measure neurophysiological correlates of creative cognition as they unfold. By simultaneously monitoring concentration and relaxation levels through α, β, and θ wave patterns during design tasks, this methodology can objectively quantify the cognitive impacts of different design tools and environments. In the present research methodology, these capabilities of EEG technology are leveraged to compare traditional versus AIGC-assisted design approaches, examining both the creative outcomes and the underlying cognitive processes that generate them. This neurophysiological perspective provides a more comprehensive understanding of how AIGC tools influence design thinking, potentially revealing cognitive mechanisms that might otherwise remain undetected through conventional assessment methods. Thus, the combined application of EEG measurement and AIGC tools creates a robust experimental paradigm for investigating the complex interactions between technology, cognition, and creative performance in design education.

## 3 Methods

### 3.1 Participants

This study was conducted at a public university in Eastern China during the Spring semester of 2024 (March to June). Participants were 64 undergraduate design students in their third year of study, recruited from the Product Design and Visual Communication Design programs using a cluster random sampling method. All participants were enrolled in a “Design Innovation” course, which focuses on developing creative problem-solving skills through project-based learning. The curriculum includes principles of human-centered design, prototyping techniques, and digital design tools.

Participants were aged between 20 and 25 years (*M* = 22.5, SD = 1.2), with similar educational backgrounds in design fundamentals and computer-aided design software. They were randomly assigned to either the experimental group (*n* = 32; 17 females, 15 males) or the control group (*n* = 32; 16 females, 16 males). All participants voluntarily took part in this study after providing written informed consent. They were fully informed of the study’s purpose and procedure and apprised of their right to withdraw at any time without penalty.

Prior to this experiment, participants had completed foundational courses in design principles, sketching techniques, and basic 3D modeling, ensuring a similar level of design knowledge and technical skills across the sample. None of the participants had prior formal training in using AIGC tools for design purposes, although most reported general familiarity with AI concepts through media exposure.

### 3.2 Research design

#### 3.2.1 Research variables

A quasi-experimental design was employed. The independent variable is the type of design tool, categorized into AIGC tools (experimental group) and traditional computer-aided design software (control group). The dependent variables are creative performance, concentration level, and relaxation level. Creative performance measures participants’ ability to generate original ideas, propose novel solutions, and produce innovative outcomes in the design task. Concentration level reflects the degree of neurophysiological engagement and sustained attention demonstrated by participants during the design process, quantified through EEG data analysis and observational measures. Relaxation level indicates the degree of psychological ease and comfort experienced by participants during the design activity. It is inversely related to the levels of stress and anxiety induced by the task and is measured through both physiological indicators and self-report measures.

#### 3.2.2 Equipment

The study was conducted in a controlled multimedia classroom environment at the university’s Design Innovation Laboratory. Each participant was equipped with a computer workstation (Dell Precision Workstation with 16GB RAM, Intel Core i7 processor, and NVIDIA GeForce RTX graphics card) connected to a 24-inch high-resolution display to ensure consistent visual experience across participants.

For EEG data collection, we utilized the BrainLink Pro headband, a commercially available portable EEG device that has been validated for research applications in educational settings (Huang et al., 2020; Liu et al., 2023). The BrainLink Pro employs a headband design with dry electrode technology that contacts the forehead, capturing signals primarily from the prefrontal cortex areas (Fp1, Fp2) according to the international 10–20 system, with a reference electrode positioned at the ear lobe (Wu and Wang, 2024). This headband design allows for comfortable, non-invasive monitoring during extended creative tasks, minimizing participant discomfort while still providing reliable EEG readings. The device captures brain electrical signals at a sampling rate of 512 Hz and transmits the data in real-time via Bluetooth to the computer for subsequent analysis using the BrainLink software platform (Version 3.5.2). This software implements proprietary algorithms for processing raw EEG signals and extracting metrics for concentration and relaxation levels based on frequency band analysis. The BrainLink Pro was selected for this study due to its established validity in attention monitoring applications, its non-invasive nature, and its ability to provide continuous recording without significant interference with the design tasks.

The EEG headband was fitted individually for each participant following a standardized protocol. Researchers first demonstrated the proper positioning, with the main sensor centered on the forehead approximately 1 inch above the eyebrows, and the reference clips attached to the earlobe. Participants were then assisted in adjusting the headband for comfort while ensuring proper sensor contact. Signal quality was verified through the software’s impedance check feature before beginning each recording session, with adjustments made until optimal signal quality was achieved (signal quality indicator showing > 85%). Prior to the main experiment, all participants underwent a 10-min familiarization session with the EEG equipment. This session also included a five-minute baseline recording during which participants were instructed to relax with eyes open while focusing on a neutral fixation cross on screen, followed by simple concentration tasks to calibrate individual baseline measurements.

For the experimental group, AIGC software tools included ChatGPT, Midjourney, and Stable Diffusion, all accessed through standardized interfaces. The control group used conventional software including Adobe Photoshop, Adobe Illustrator, Rhinoceros, KeyShot, and Autodesk Maya, with all participants receiving equivalent training in software operation prior to the experiment to minimize variability due to differing software proficiency levels. The EEG device processes raw brain electrical signals through several automated steps to generate the concentration and relaxation scores used in the present analysis. First, the raw EEG signals captured at 512 Hz undergo preprocessing to filter out noise and artifacts. The cleaned signals are then decomposed into standard frequency bands (α, β, and *θ*) using Fast Fourier Transform. The eSense algorithm calculates concentration scores based on the ratio of β waves (13–30 Hz, associated with focused attention) to θ waves (4–7 Hz, associated with distraction), while relaxation scores are derived from α wave power (8–12 Hz, associated with calm states) relative to high β activity (>30 Hz, associated with stress). These raw metrics are normalized to a 0–100 scale using manufacturer-calibrated population norms, where higher values indicate greater concentration or relaxation. Throughout the three-hour design task, scores are calculated continuously using one-second time windows, with the final analysis using the mean values for each participant. This standardized processing ensures objective, comparable measurements of neurophysiological states across all participants.

#### 3.2.3 Research procedure

**Figure 1:**
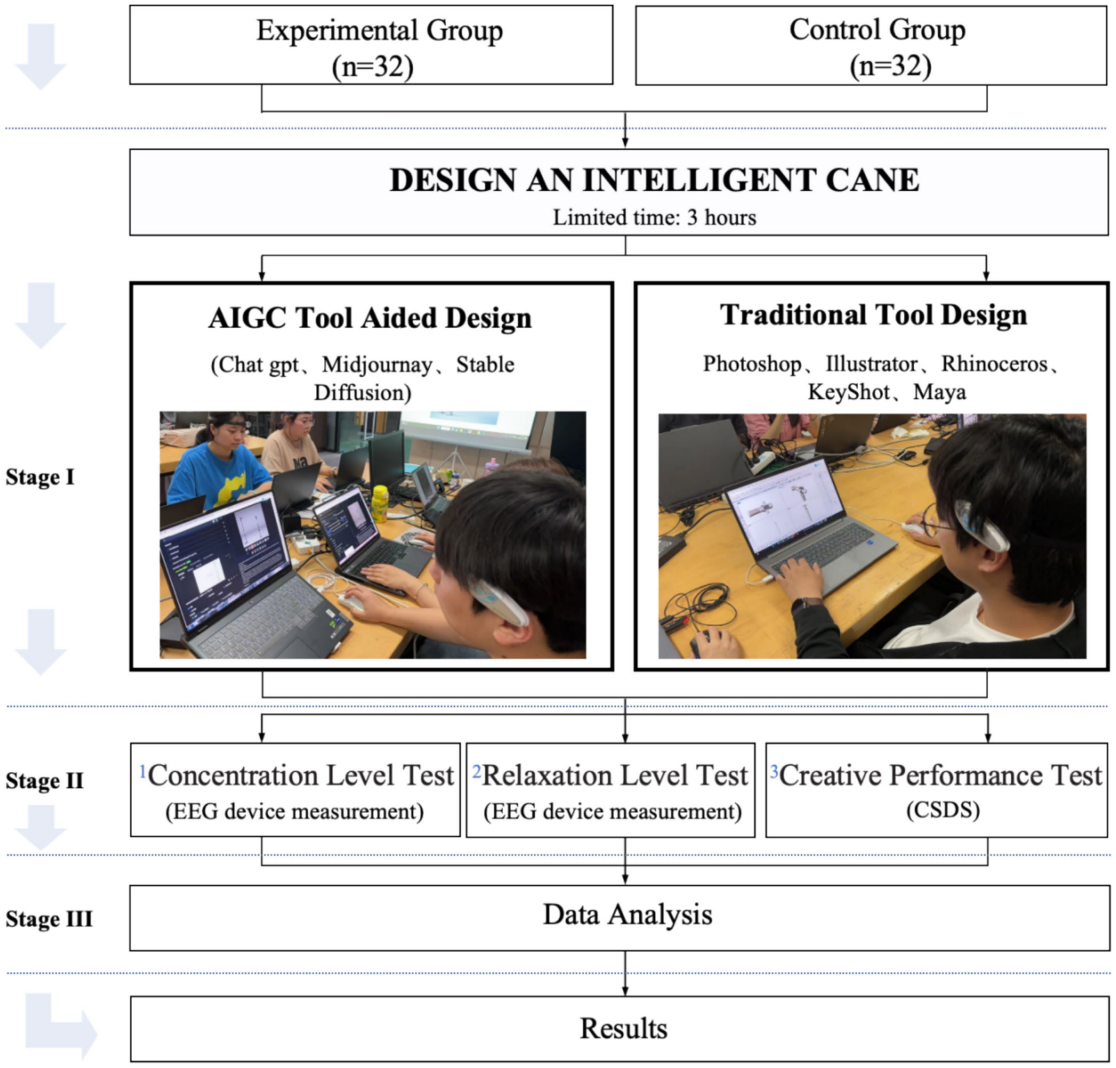
Research procedure.

This study was approved by the ethics committee of the first author’s institution prior to commencement. Figure 1 illustrates the research process. The experimental group and control group were arranged in separate rooms, with each group accommodating a maximum of five participants simultaneously. Participants were assigned the task of designing an intelligent cane. Throughout the entire design process, both groups of participants wore portable EEG devices. These devices continuously monitored and recorded the participants’ concentration and relaxation levels. Upon completion of the design task, three independent raters evaluated the participants’ works using the creative solution diagnosis scale (CSDS) to derive creative performance scores. Subsequently, to understand participants’ subjective experiences, we designed a semi-structured interview questionnaire. The interview content covers participants’ perceptions of the design process, software experiences, challenges encountered, and satisfaction with the outcomes. After the completion of the design task, a university lecturer conducted 30-min interviews with participants, encouraging them to freely express their genuine thoughts.

### 3.3 Instruments

#### 3.3.1 Creative performance test

To assess participants’ creative performance, this study employed the Creative Solution Diagnosis Scale (CSDS) developed by Cropley (2021). The CSDS is a 30-item Likert-type scale utilizing a five-point rating system, designed to evaluate product creativity across multiple dimensions. This scale is based on four core dimensions of creative performance: Relevance and Effectiveness (fit to task requirements and practical value), Novelty (uniqueness and innovativeness), Elegance (esthetic harmony and sophistication), and Genesis (originality, inspirational quality, and ability to break conventional thinking patterns).

Participants’ creative works were independently scored by three design field experts using the CSDS. Each item was scored on a scale from 1 (strongly disagree) to 5 (strongly agree). Dimension scores were calculated using weighted averages, with higher scores (out of a possible 150) indicating better creative performance. The CSDS has demonstrated robust psychometric properties, reporting high internal consistency (Cronbach’s α = 0.84–0.89) and strong inter-rater reliability (ICC > 0.82).

#### 3.3.2 EEG measurement

EEG equipment was used to objectively measure participants’ concentration and relaxation levels during the design task process in this research. The device employs dry electrode technology, collecting EEG signals from the prefrontal (Fp1, Fp2) and temporal (T3, T4) regions according to the international 10–20 system, with a sampling rate of 512 Hz. The device uses a proprietary eSense algorithm for real-time signal processing and analysis. This algorithm has been validated in multiple studies, including applications in attention assessment and emotion recognition tasks (Liu et al., 2023).

The eSense algorithm first preprocesses the raw EEG signals, using digital filtering techniques to remove environmental noise and muscle interference. It then extracts key features reflecting cognitive and emotional activities, such as energy or power spectral density in different frequency bands (Białas et al., 2022). The algorithm focuses on analyzing several key brainwaves: (i) β waves are closely related to concentration level, with increased activity typically indicating higher alertness and focused attention; (ii) *θ* waves are negatively correlated with concentration level, with increased activity indicating distraction or drowsiness (Arnau-González et al., 2021). (iii) α waves are closely related to relaxation level, with increased activity typically indicating a more relaxed state; (iv) while enhanced high β waves (>30 Hz) indicate increased levels of stress or anxiety (Niso et al., 2023).

Based on these features, the eSense algorithm calculates continuous values (0–100) for concentration and relaxation levels using a 1-s sliding time window. Higher concentration levels indicate better focus, while higher relaxation levels suggest a more relaxed state (Huang et al., 2020). These real-time values are transmitted via Bluetooth to the accompanying software, which calculates and displays mean levels after task completion. These means serve as overall indicators of each participant’s performance and are used for subsequent analysis.

### 3.4 Task-oriented practical project

#### 3.4.1 Test design task

The design task in this study requires participants to comprehensively design an intelligent walking cane for the elderly or individual with mobility impairments within 3 h. The design should incorporate multiple dimensions, including but not limited to: functionality, intelligent features, ergonomic considerations, esthetic appeal, technological innovation, and sustainable durability. During the design process, participants need to develop a deep understanding of the characteristics and needs of the target population. Participants are required to submit design sketches, three-dimensional models, and rendered images as deliverables to fully demonstrate their design concepts and process. Participants are encouraged to employ innovative thinking, proposing novel design concepts and solutions to comprehensively meet the needs of the target individuals.

#### 3.4.2 Products designed by the experimental group

Researchers observed the experimental group participants’ design process, which utilized ChatGPT for concept ideation and creative exploration. Participants input design task details and inquired about potential functions, interaction methods, and technological approaches for the intelligent walking cane. ChatGPT provided design inspiration, reference cases, and optimization suggestions based on its knowledge base. Through multiple rounds of dialog, participants clarified the core concepts and key features of the design. Subsequently, participants used Stable Diffusion to generate creative intelligent walking cane design images based on prepared prompts. They optimized the results by adjusting parameters such as resolution, iteration count, and artistic style. Finally, they used Photoshop or Illustrator for post-processing and layout. Most experimental group participants completed the entire process from concept ideation to image generation and layout within 3 h.

**Figure 2:**
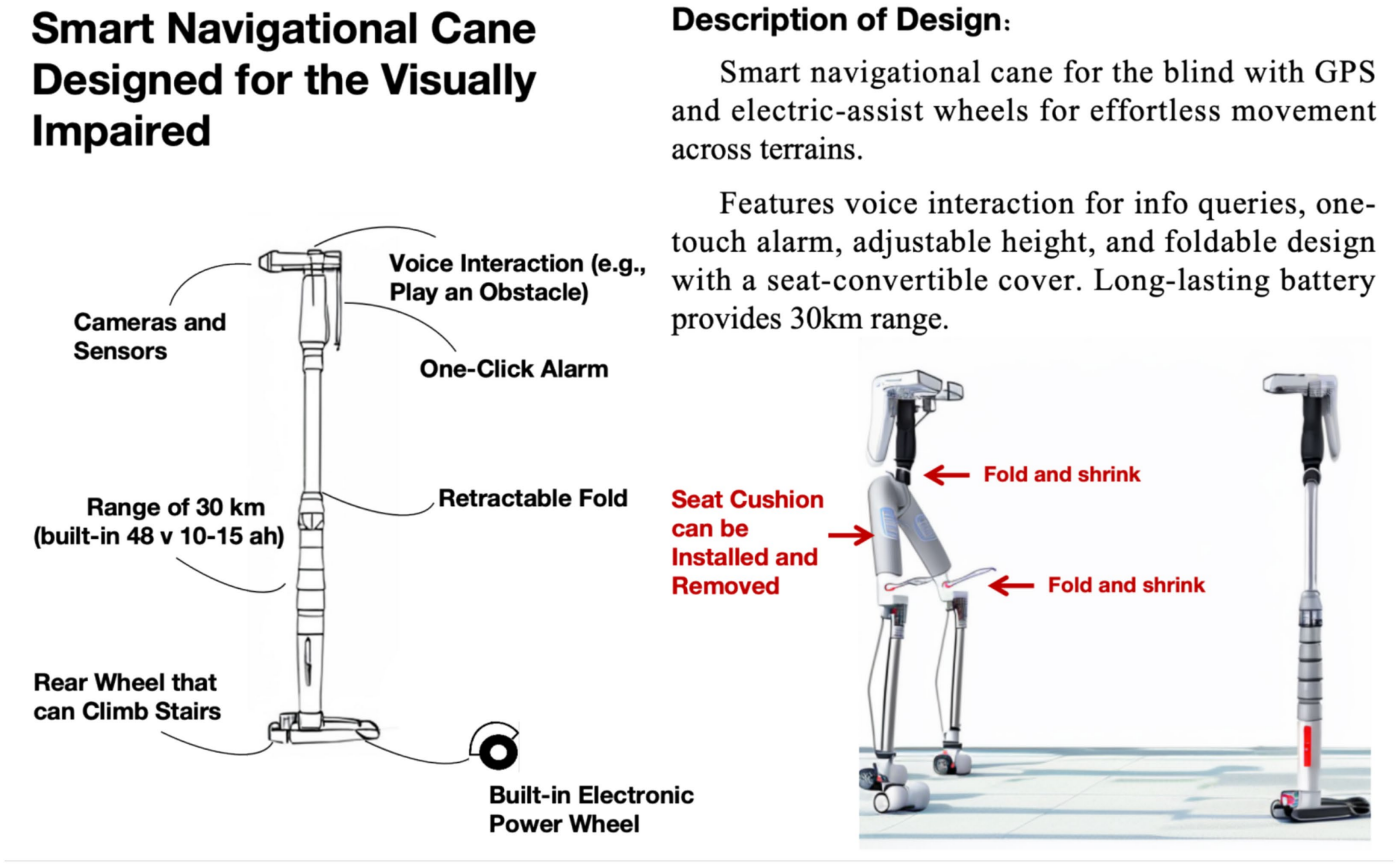
Work by a participant in the experimental group.

Figure 2 illustrates an intelligent navigation cane designed by an experimental group participant for visually impaired individuals, conceived after discussions with ChatGPT on topics such as visual impairment, navigation, and interaction design. This multifunctional cane integrates intelligent navigation, assisted walking, and voice interaction capabilities. It features a camera and sensors for environmental perception, voice interaction for providing navigation information and environmental descriptions, and a one-key alarm function for enhanced safety. The design incorporates built-in electronic power-assisted wheels that adapt to various road conditions, including stairs, and a foldable structure with a detachable seat pad for portability and temporary rest. Powered by a 48 V, 10–15 Ah lithium-ion battery, the cane offers an operational range of approximately 30 kilometers, sufficient for typical daily use.

#### 3.4.3 Products designed by the control group

The control group participants employed traditional design methods without the assistance of AIGC tools. Their design process encompassed several stages: First, participants conducted preliminary research by searching and filtering relevant literature and existing smart cane products via the internet. They then visualized their initial concepts as conceptual sketches using either hand-drawn techniques with pen and paper or computer-aided design software. After sketching, participants utilized 3D modeling software such as Rhinoceros to transform their 2D concepts into 3D digital models. They generated high-quality product visualizations using rendering software like KeyShot. Finally, participants employed image processing software such as Photoshop and Illustrator to perform post-production optimization and layout design on their rendered works, including color adjustment, background enhancement, and the addition of textual descriptions and annotations to elucidate the design features and functions of the product.

**Figure 3:**
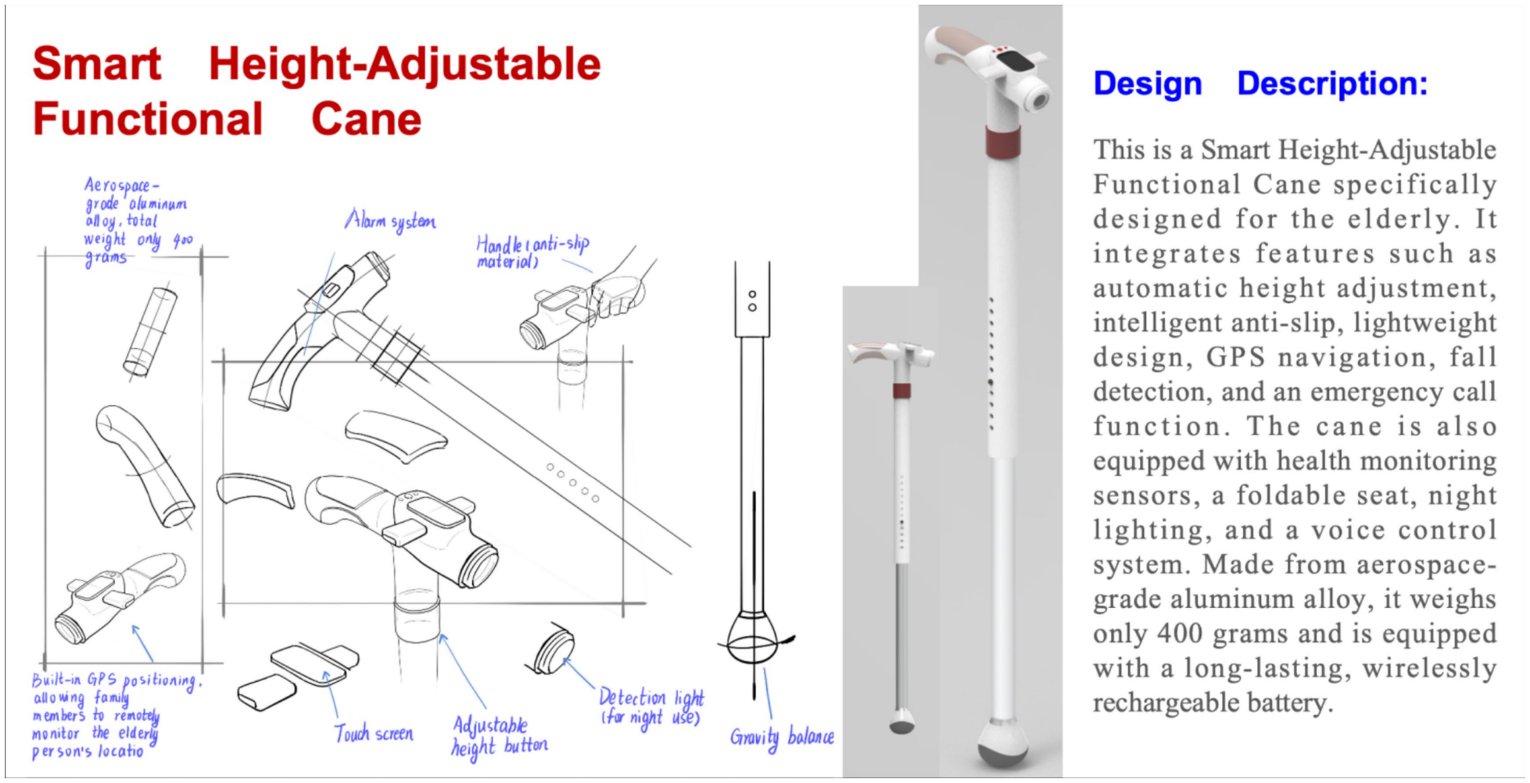
Work by a participant in the control group.

Figure 3 illustrates an intelligent cane designed by a participant from the control group. Based on collected data, the researcher identified that elderly and disabled individuals frequently face challenges with inappropriate cane height and a high risk of slipping. Consequently, they developed this cane with automatic height adjustment and enhanced traction features. The device incorporates height sensors, an electric telescoping mechanism, high-friction materials, and pressure sensors that activate LED warning indicators when detecting slippery surfaces. This smart fall-prevention cane for the elderly also integrates GPS navigation, fall detection, emergency alert system, health monitoring capabilities, a foldable seat, night illumination, and voice control functionality. Constructed from aviation-grade aluminum alloy, the device weighs 400 grams and is powered by a high-capacity, wirelessly rechargeable battery. This innovative assistive tool aims to enhance safety, independence, and quality of life for elderly users by combining advanced technology with ergonomic design principles.

### 3.5 Sample size justification

The sample size of 32 participants per group (total N = 64) was determined through *a priori* power analysis using G*Power 3.1.9.7 software. Based on previous research examining the effects of AI-assisted design tools on creative performance (Urban et al., 2024; Wang S. et al., 2023), a medium effect size was anticipated for between-group differences (Cohen’s d = 0.5). With a significance level of α = 0.05, desired statistical power of 0.80, and using a two-tailed independent samples t-test, the required sample size was calculated to be 64 participants (32 per group). This sample size provides sufficient statistical power (80%) to detect significant between-group differences while also accounting for potential data loss or participant attrition.

### 3.6 Data analysis

All statistical analyses were conducted using SPSS version 28.0 (IBM Corp., Armonk, NY, United States). Prior to conducting the main analyses, data normality was assessed using the Shapiro–Wilk test due to the moderate sample size (*n* = 64). Results confirmed that all dependent variables met normality assumptions (*p* > 0.05). Levene’s test verified homogeneity of variance between groups (*p* > 0.05), satisfying the prerequisites for parametric testing. The analytical approach included descriptive statistics, independent samples t-tests for between-group comparisons, and Pearson correlation analysis for examining variable relationships. Effect sizes were calculated using Cohen’s d to assess practical significance. Statistical significance was set at α = 0.05. Qualitative interview data underwent thematic analysis following Jiang et al. (2024)’s framework, with independent coding by two researchers to ensure analytical reliability.

### 3.7 Quantitative analysis results

Table 1 presents the descriptive statistics for the independent and dependent variables. The study included a total of 64 participants, with 32 in the experimental group and 32 in the control group. The analysis indicates that the experimental group demonstrated higher mean scores in creative performance, concentration, and relaxation compared to the control group. Specifically, the experimental group’s mean creative performance score was higher (*M* = 115.13) than that of the control group (*M* = 110.69). For concentration levels, the experimental group scored higher (*M* = 51.06) than the control group (*M* = 48.31). Similarly, the experimental group showed higher relaxation levels (*M* = 48.59) compared to the control group (*M* = 47.84).

**Table 1:** Descriptive statistics for creative performance, concentration level, and relaxation level by group.

	Modes	Mean	Std. Deviation	*N*
Creative performance	Experimental group	115.13	6.440	32
	Control group	110.69	9.372	32
	Total	112.91	8.284	64
Concentration level	Experimental group	51.06	2.539	32
	Control group	48.31	2.867	32
	Total	49.69	3.023	64
Relaxation level	Experimental group	48.59	2.284	32
	Control group	47.84	2.259	32
	Total	48.22	2.285	64

The results of independent samples t-tests are presented in Table 2. The analysis revealed a statistically significant difference between the two groups in creative performance scores [*t*(62) = 2.208, *p* = 0.031]. Similarly, concentration level also showed a statistically significant between-group difference [*t*(62) = 4.062, *p* < 0.001]. However, for relaxation level, no statistically significant difference was observed between the two groups [*t*(62) = 1.321, *p* = 0.191].

**Table 2:** Results of independent samples *t*-tests.

Variables	df	*t*	Sig. (2-tailed)	Mean difference	95% confidence interval of difference	
					Lower	Upper
Creative performance	2.208	62	0.031	4.438	0.419	8.456
Concentration level	4.062	62	0.000	2.750	1.397	4.103
Relaxation level	1.321	62	0.191	0.750	−0.385	1.885

**Figure 4:**
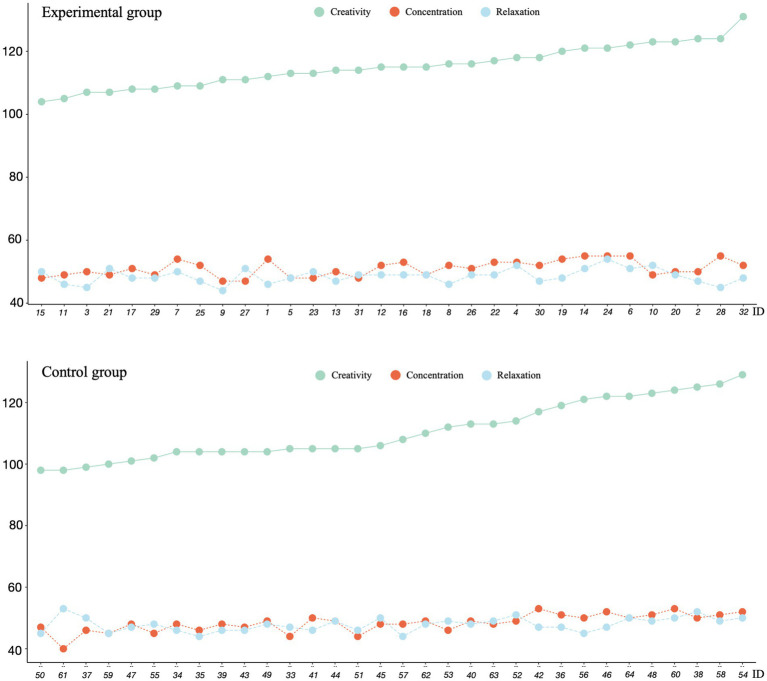
Multivariate dot plot of groups.

To visualize individual differences and relationship patterns beyond group means, the distribution of scores across all participants was examined. The individual data patterns reveal interesting relationships between creative performance and neurophysiological states that may not be apparent from group-level statistics alone. Figure 4 presents the creative performance, concentration level, and relaxation level scores for each participant in the experimental and control groups. The multivariate dot plot reveals a positive correlation between creative performance and concentration levels. Participants with higher creative performance scores also generally exhibited higher concentration levels. This relationship appears to be more pronounced in the experimental group. The relationship between creative performance and relaxation levels was less consistent, though occasionally positive. This might imply that moderate relaxation level is beneficial for creative performance, but excessive relaxation level may not further enhance creative performance. Moreover, there appears to be a positive correlation between concentration and relaxation levels. For most participants, the scores for these two indicators are close and follow similar trends. This might suggest that participants achieved a state of psychological balance while performing creative tasks, maintaining sufficient attention without being overly tense.

**Figure 5:**
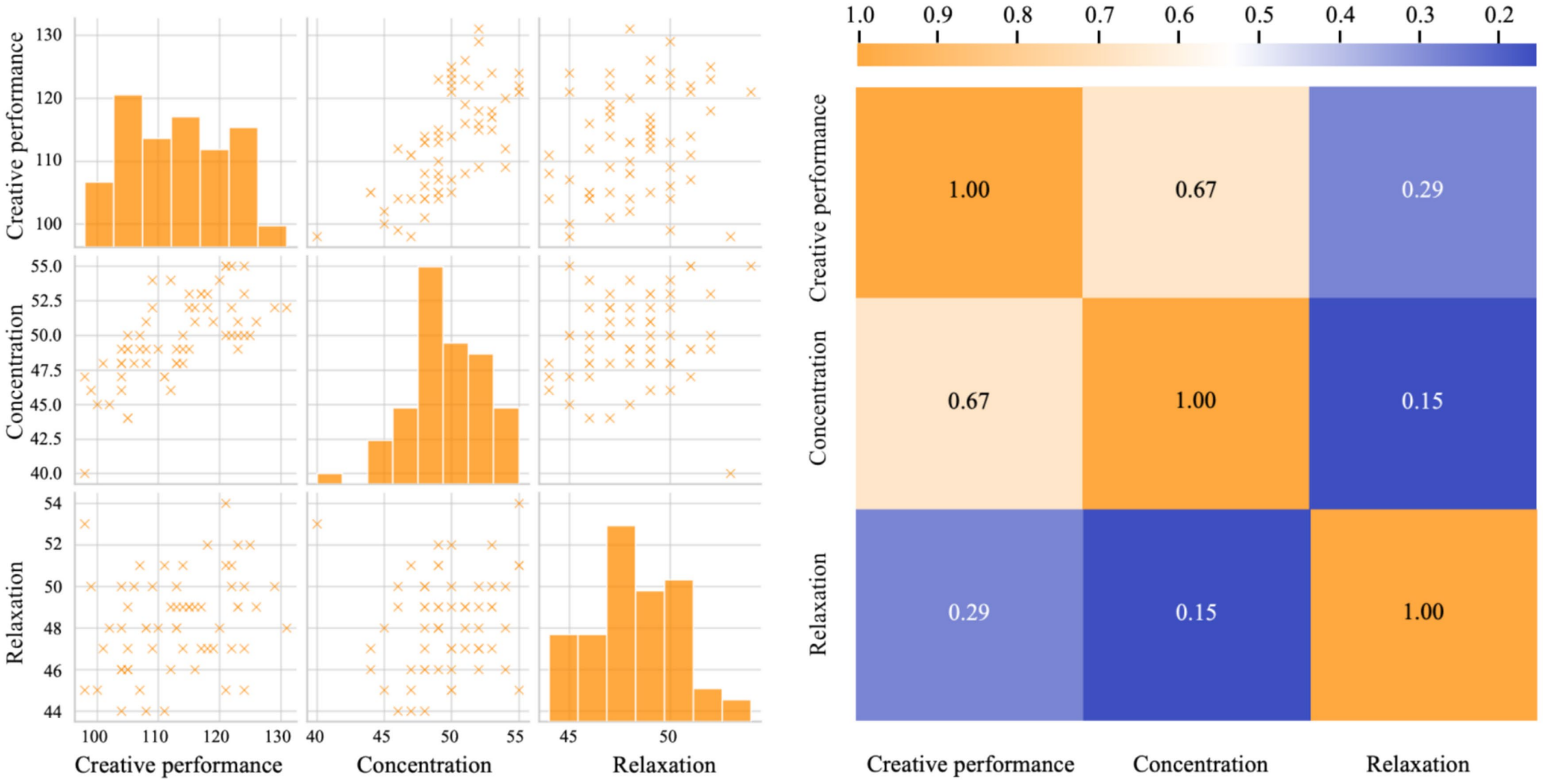
Scatter plot correlation matrix of dependent variables.

To further explore the relationships among the variables, a scatter plot correlation matrix was created. As illustrated in Figure 5, it indicates a moderate positive linear relationship between creative performance and concentration level (*r* = 0.67, *p* < 0.001), as evidenced by the clustered upward trend in the scatter plot. This finding suggests that individuals exhibiting higher creative performance also tend to show elevated concentration scores. In contrast, the relationships between relaxation level and both creative performance (*r* = 0.29, *p* = 0.021) and concentration level (*r* = 0.15, *p* = 0.237) appear weaker. The scatter plots for these pairs display a more dispersed distribution of data points, with only the relaxation-creative performance correlation reaching statistical significance. The histograms further highlight the distinct distribution characteristics of each variable, with creative performance exhibiting a relatively uniform spread, concentration level displaying a slight right skew, and relaxation level presenting a near-normal distribution.

To further explore the relationships among the variables, a scatter plot correlation matrix was created. As illustrated in Figure 5, it indicates a moderate positive linear relationship between creative performance and concentration level (*r* = 0.67, *p* < 0.001), as evidenced by the clustered upward trend in the scatter plot. This finding suggests that individuals with higher creative performance also tend to show elevated concentration scores. In contrast, the relationships between relaxation level and both creative performance (*r* = 0.29, *p* = 0.021) and concentration level (*r* = 0.15, *p* = 0.237) appear weaker.

The histograms reveal notable distribution characteristics that inform the interpretation of the results. Creative performance scores display a relatively widespread across the range (100–130), suggesting considerable individual variation in creative outcomes regardless of the design tools used. This broad distribution indicates that while AIGC tools significantly improved mean performance, individual differences in creative ability remained substantial. The concentration level histogram shows a near-normal distribution centered around 48–52, indicating that most participants maintained moderate to high attention levels during the design task. This pattern suggests a consistent level of neurophysiological engagement across participants, with the AIGC group shifting the entire distribution toward higher values rather than creating extreme outliers. The relaxation level also presents a near-normal distribution (centered around 48–50), indicating that most participants maintained moderate relaxation states during the design task, with relatively few experiencing either high stress or deep relaxation. This finding supports the interpretation that AIGC tools do not fundamentally alter stress levels during creative work, but rather redirect cognitive resources toward more focused attention. Together, these distribution patterns suggest that AIGC’s benefits operate primarily through optimizing attention allocation rather than reducing task-related stress or homogenizing creative abilities. These correlational findings should be interpreted with caution, as correlation does not imply causation. While concentration and creative performance are strongly associated, the directionality of this relationship cannot be determined from correlational analysis alone.

### 3.8 Qualitative analysis results

To complement the quantitative findings, qualitative data from semi-structured interviews were analyzed using thematic analysis. Interviews were conducted immediately after each participant completed the design task, led by a trained psychology teacher who was unaware of the participants’ group assignments. The interviews lasted approximately 30 min and explored participants’ subjective experiences and cognitive processes during the design task. Table 3 presents a comparative summary of the key themes that emerged from the interviews, highlighting the differences between the experimental (AIGC-assisted) and control (traditional tools) groups across several dimensions of the design experience.

**Table 3:** Comparative thematic analysis of semi-structured interviews.

Themes	Experimental group (AIGC)	Control group (traditional)
Concentration state	High levels of focus on conceptual aspects; “ChatGPT allowed me to maintain deep concentration on core design concepts rather than technical details” (P7); “Using AIGC tools helped me stay focused for longer periods without mental fatigue” (P15)	More fluctuating attention patterns; “I found myself switching between different software constantly, which disrupted my concentration” (P39); “Rendering processes required intense focus but also created frustrating waiting periods” (P44)
Design idea development	Rapid ideation and iteration; “Midjourney allowed me to quickly transform abstract concepts into concrete visual effects, greatly accelerating the design iteration process” (P11); “AI tools helped me explore multiple design directions simultaneously” (P23)	More linear progression with fewer iterations; “I spent considerable time in the early planning stage before committing to a design direction” (P37); “Hardware limitations restricted how many design alternatives I could fully develop” (P52)
Creative process experience	Enhanced creative confidence with occasional unpredictability; “ChatGPT acted like a catalyst for my thoughts, effectively expanding my design ideas” (P3); “AI generated unexpected suggestions when I hit bottlenecks” (P18)	Greater sense of control but more creative challenges; “I felt more personal ownership of every design decision” (P41); “Sometimes I felt stuck in a creative rut, struggling to generate truly novel ideas” (P48)
Technical challenges	Content quality management; “AI-generated content is sometimes too eccentric, requiring extra time for screening and adjustment” (P5); “The content generated based on my instructions differed from the expected results” (P29)	Software proficiency limitations; “Hardware limitations led to time-consuming rendering processes” (P38); “I struggled with complex modeling techniques required for my design vision” (P55)
Time management	Efficiency in execution; “AIGC directly helped me generate the final design work, saving significant time” (P12); “I could allocate more time to evaluating and refining concepts rather than creating initial drafts” (P25)	Resource allocation challenges; “Spending too much time reviewing literature and materials left little time for the later design phase” (P36); “The technical execution took longer than anticipated” (P49)
Perceived control	Partnership with technology; “Working with AI felt like having a collaborative partner to bounce ideas off” (P14); “I directed the AI while it handled technical aspects, I’m not proficient in” (P19)	Complete authorship; “I was able to precisely control every design detail” (P45); “The final product feels entirely my own creation” (P60)

Analysis of the interview data revealed distinct differences in how participants from each group experienced the design process. The experimental group generally reported higher efficiency, more rapid ideation cycles, and enhanced creative confidence when using AIGC tools. These participants described their concentration as being redirected from technical details to higher-level conceptual thinking. In contrast, control group participants experienced more direct control over their design process but faced greater challenges with creative bottlenecks and technical limitations. Their concentration patterns tended to be more fragmented as they navigated between different software tools and technical processes.

The qualitative findings align with and provide context for the quantitative results, particularly regarding the higher concentration levels observed in the experimental group. The interviews suggest that AIGC tools facilitated a shift in cognitive resource allocation, allowing participants to maintain sustained attention on conceptual aspects of design while reducing cognitive load associated with technical execution. This qualitative context helps explain why AIGC tools led to improvements in both creative performance and concentration levels, despite not significantly affecting overall relaxation levels.

## 4 Discussion

### 4.1 Creative performance differences between AIGC and traditional design approaches

The experimental results demonstrated significant creative performance advantages for AIGC-assisted design, with participants achieving higher scores (*M* = 115.13, SD = 6.440) compared to traditional design approaches (*M* = 110.69, SD = 9.372), *t*(62) = 2.208, *p* = 0.031, Cohen’s d = 0.55. This medium-to-large effect size aligns with recent findings showing similar creative performance improvements in university students using ChatGPT for creative problem-solving, though previous research focused on text-based rather than visual-design tasks Urban et al. (2024). These findings extend this research by demonstrating that AIGC benefits translate effectively to visual design domains with measurable creative output improvements.

The creative performance enhancement appears to operate through multiple interconnected mechanisms that build upon existing creativity theories while revealing new aspects of AI-human creative collaboration. First, AIGC tools function as cognitive amplifiers that break conventional ideation boundaries, supporting findings on AI’s capacity to expand creative interpretation of visual stimuli Grassini and Koivisto (2024). The results show that human-AI collaboration can enhance human creativity through complementary rather than competitive interactions. The interview data revealed that participants experienced what can be characterized as “conceptual liberation” - the ability to explore design territories that might remain inaccessible through traditional approaches alone. As one participant articulated: “AI generated unexpected suggestions when I hit bottlenecks” (P18), indicating that AIGC assistance provides alternative cognitive pathways when conventional thinking reaches impasses. This mechanism aligns with dynamic network theory, where creative breakthroughs occur through novel combinations of disparate cognitive networks, extending it by showing how AI can facilitate these network interactions (Beaty et al., 2018).

Second, the performance enhancement stems from accelerated iteration-evaluation cycles that compress traditional design timelines while expanding explorative breadth. This finding supports research identifying iteration acceleration as a key benefit of AI design tools, though previous studies focused on collaborative team dynamics rather than individual cognitive processes (Lee et al., 2024). The neurophysiological data provide objective evidence for these temporal efficiency gains, showing sustained concentration levels (*M* = 51.06) that enable deeper engagement with creative tasks. Participants described completing “the entire process from concept ideation to image generation and layout within 3 h” with significantly more design variations explored compared to traditional approaches. This temporal efficiency enables what can be termed “iterative deepening”—the ability to cycle rapidly between generation and evaluation phases, allowing for deeper conceptual refinement within limited timeframes. Furthermore, AIGC assistance enables cognitive resource reallocation from technical execution toward higher-order creative evaluation, supporting findings on cognitive load redistribution in AI-assisted environments (Dalilian and Nembhard, 2024). However, while their research focused on error detection tasks, this study demonstrates similar cognitive reallocation effects in creative contexts. Participants reported spending substantially more time on conceptual assessment and refinement activities, as evidenced by statements like “I could allocate more time to evaluating and refining concepts rather than creating initial drafts” (P25). This redistribution suggests that AIGC tools address what can be identified as the “technical bottleneck problem” in design education—where students’ creative potential is constrained by technical skill limitations rather than conceptual capacity.

The findings both support and provide different perspectives on existing literature regarding AI-assisted creativity. While previous research reported enhanced creative self-efficacy in AI-supported educational environments (Wang S. et al., 2023), this study provides neurophysiological evidence for the cognitive mechanisms underlying these improvements. The results show a different pattern from research expressing concerns about AI inhibiting independent creative thinking (Magni et al., 2024). Instead, the data indicate that creative performance improvements were accompanied by enhanced concentration levels, suggesting active neurophysiological engagement rather than passive dependence. This difference may reflect variations in task complexity and measurement approaches, highlighting the need for multi-dimensional assessments of AI’s impact on creativity. However, the creative performance enhancement requires careful interpretation within educational contexts. While the quantitative improvements are substantial, the nature of creativity measurement itself presents complexities. The CSDS scale, though validated, captures specific dimensions of creative output that may not encompass all aspects of creative development essential for design education. The observed improvements may reflect enhanced creative productivity rather than fundamental creative capacity development, raising questions about long-term creative skill acquisition and independence that warrant longitudinal investigation.

### 4.2 Neurophysiological state modulation through technology-mediated design processes

The neurophysiological measurements revealed differential impacts of AIGC tools on concentration and relaxation states, providing objective evidence for technology-induced neurophysiological state changes during creative work. Concentration levels showed significant enhancement under AIGC conditions (*M* = 51.06, SD = 2.539) compared to traditional design approaches (*M* = 48.31, SD = 2.867), *t*(62) = 4.062, *p* < 0.001, Cohen’s d = 1.02, representing a large effect size with substantial practical implications for attention management during creative tasks. These findings complement recent EEG studies on attention enhancement in virtual reality creative environments, though this study extends the research to AI-assisted design contexts with portable EEG monitoring (Wu et al., 2024).

The concentration enhancement mechanism appears linked to what can be termed “cognitive scaffolding effects” where AIGC tools provide structured support that enables sustained attention allocation. This aligns with cognitive load theory frameworks established by Sweller and colleagues, though the application to AI-assisted creativity represents a novel extension. Interview data revealed that participants experienced “high levels of focus on conceptual aspects” where “ChatGPT allowed me to maintain deep concentration on core design concepts rather than technical details” (P7). This cognitive redistribution supports findings on attention management in technology-enhanced learning, though the neurophysiological evidence provides more objective measurement of these attention effects than previous self-report studies (Chen et al., 2017). The sustained concentration improvements show a different pattern from findings reporting attention fluctuations in VR creative environments (Huang and Chang, 2023). This difference may reflect the nature of AI assistance versus immersive virtual environments - while VR can create attention fragmentation through sensory overload, AIGC tools appear to provide attention focusing through cognitive support rather than sensory immersion. The neurophysiological evidence supports this interpretation, as the sustained higher concentration levels suggest more efficient attention allocation rather than simply increased mental effort.

Conversely, relaxation levels showed no significant difference between AIGC (*M* = 48.59, SD = 2.284) and traditional design conditions (*M* = 47.84, SD = 2.259), *t*(62) = 1.321, *p* = 0.191. This finding shows a different pattern from research documenting cognitive load reduction in AI-assisted tasks, though it aligns with complex findings on relaxation states during creative activities (Dalilian and Nembhard, 2024; Liu et al., 2021). The maintained relaxation levels despite enhanced concentration suggest that AIGC tools create what can be characterized as “balanced cognitive activation”—heightened attention without corresponding stress or anxiety increases. The findings on stable relaxation levels also contribute to ongoing debates in creativity research about the role of relaxation in creative processes. While some research emphasized relaxation’s importance for creative thinking, and others showed positive correlations between relaxed states and creative performance, these results suggest a more complex relationship (Kora et al., 2021). The stable relaxation levels in both conditions, combined with differential concentration patterns, indicate that optimal creative performance may depend more on attention management than stress reduction. This provides a different perspective on the dynamic relationship between attention states and creative thinking, with the AI-assisted context adding new dimensions to these relationships (Carruthers et al., 2018).

The interview data provide deeper insights into this neurophysiological balance that build upon existing literature while revealing new patterns. AIGC participants reported experiencing simultaneous neurophysiological engagement and creative comfort, describing the AI interaction as “like having a collaborative partner to bounce ideas off” (P14). This finding extends theoretical frameworks on human-AI co-creative systems by providing neurophysiological evidence for the psychological comfort these systems can provide (Rezwana and Maher, 2023). The results also reveal complexity not fully addressed in previous frameworks - participants noted that AI-generated content sometimes required “extra time for screening and adjustment” (P5), suggesting that cognitive trade-offs exist that maintain overall activation levels even when specific cognitive loads are reduced.

### 4.3 Neurophysiological-creative performance relationships and their moderation by design technology

The correlation analysis revealed complex relationships between neurophysiological states and creative performance that were moderated by design technology conditions, extending existing literature on creativity-cognition relationships into AI-assisted contexts. Across both conditions, concentration levels demonstrated strong positive correlations with creative performance (*r* = 0.67), supporting the established findings of Krauzlis et al. (2023) regarding attention’s central role in complex cognitive tasks. However, relaxation levels showed weaker associations (*r* = 0.29), partially contradicting Liu et al. (2021), who emphasized a stronger role of relaxation in creative thinking. This discrepancy may reflect task-specific differences, as the present design task required sustained problem-solving that favors concentrated attention over relaxed states.

The relationship patterns differed between AIGC and traditional design conditions in ways that both support and extend existing theoretical frameworks. In traditional design environments, the concentration-performance correlation was stronger and more linear, aligning with controlled processing theories established by Schneider and Shiffrin, where complex cognitive tasks require deliberate, resource-intensive mental operations. Participants using traditional tools described needing to “precisely control every design detail” (P45), indicating high cognitive control demands throughout the design process. This supports Nguyen et al.’s (2018) findings on cognitive effort requirements in conceptual design processes, though our EEG measurements provide more precise neurophysiological evidence than their behavioral observations.

In contrast, AIGC-assisted design conditions showed slightly attenuated concentration-performance correlations, accompanied by enhanced overall performance levels. This pattern builds upon recent research by Hanafy (2023) concerning the influence of AI on design creativity, by providing neurophysiological evidence of altered cognitive processing patterns. The finding suggests that AIGC tools enable creative success through more diverse cognitive pathways, reducing reliance on pure attentional control while maintaining creative effectiveness. This supports emerging theories of distributed cognition in human-AI systems, though the present study provides the first neurophysiological evidence for these theoretical propositions in creative contexts. The relationship between relaxation and creative performance remained consistently weak across both conditions (*r* = 0.29), but manifested differently in each environment in ways that contribute new insights to creativity research. Traditional design participants described relaxation periods as necessary recovery phases between intense technical work sessions, supporting established models of creative process cycling. However, AIGC participants experienced more continuous moderate relaxation throughout the design process, suggesting that AI assistance may enable what can be term “relaxed engagement”—a neurophysiological state where creative work proceeds without excessive stress while maintaining productive output levels. This finding challenges binary models of creative cognition that emphasize either focused attention or relaxed states, supporting more recent dynamic models proposed by Yang et al. (2024).

The moderate positive correlation between concentration and relaxation levels (*r* = 0.15) provides additional insights that extend existing literature on optimal neurophysiological states for creative work. While previous research often treated concentration and relaxation as opposing states, the findings suggest they can be synergistic under appropriate technological conditions. This relationship was more pronounced in the AIGC condition, where participants achieved simultaneous attention enhancement and stress management. This supports and extends Wu and Wang’s (2024) research on balancing cognitive control and spontaneous thinking, but the present neurophysiological evidence provides objective measurement of these balance states rather than relying on behavioral indicators alone. The finding also aligns with emerging neuroscience research on creative cognition that emphasizes dynamic network interactions rather than static neurophysiological states.

## 5 Conclusion

This study employed EEG technology to investigate the cognitive and creative impacts of AIGC tools in design education, revealing significant insights into the intersection of artificial intelligence and human creativity. The findings demonstrate that AIGC tools substantially enhance both creative performance and concentration levels among design students, suggesting that AI-assisted design environments can effectively optimize cognitive resource allocation and creative output quality. The relationship between neurophysiological states and creative performance presents a nuanced picture, with concentration levels showing strong correlations with creative outcomes while relaxation levels exhibit weaker associations. This pattern indicates that AIGC tools may fundamentally alter the cognitive dynamics of creative work, shifting emphasis from traditional relaxation-dependent ideation to more focused, concentration-intensive creative processes. Such cognitive reconfigurations suggest that AI-assisted creativity operates through different neurophysiological pathways than conventional creative approaches, with AIGC tools demonstrating their value through multiple mechanisms: cognitive enhancement that disrupts established thinking patterns, accelerated feedback cycles that enable rapid iteration and refinement, and emotional stimulation that boosts creative confidence and motivation.

While concerns about creative dependency and skill development warrant attention, these challenges represent opportunities for educational innovation rather than fundamental limitations. The key lies in developing pedagogical approaches that harness AI’s creative augmentation capabilities while simultaneously fostering students’ independent thinking and core design competencies, ensuring that AIGC serves as a catalyst for human creativity rather than a substitute. The integration of EEG measurement with AIGC-assisted design provides a robust framework for understanding creative cognition in technology-enhanced environments, opening new avenues for optimizing AI-human creative collaboration, developing adaptive learning systems, and creating more effective design education practices. As AI technologies continue to evolve, this research contributes valuable insights for educators seeking to maximize the creative potential of both human learners and artificial intelligence systems working in partnership, ultimately expanding the boundaries of what students can achieve in creative domains.

## 6 Limitation and further research

This study has several limitations that warrant consideration. The sample of 64 participants was limited to design students from a single university in Eastern China, which may restrict the generalizability of findings across different cultural contexts, educational systems, and academic disciplines. Future research should conduct multi-institutional studies across diverse geographical regions and cultural contexts to establish broader applicability and cross-cultural validity of AIGC effects on creative performance. The study focused on a single 3-h design session, which may not capture the long-term effects of AIGC tools on students’ creative development, potential dependency issues, or skill retention. Longitudinal studies tracking students’ creative abilities, tool dependency patterns, and independent thinking skills over extended periods (e.g., semester-long or multi-year studies) would provide more comprehensive insights into the sustained impacts of AIGC integration in design education. Although EEG technology provided objective measurements of neurophysiological states, the single neuroimaging approach may not fully capture the complexity of creative cognitive processes. Future research could benefit from combining EEG with complementary neuroimaging techniques (such as fMRI for spatial resolution) or physiological indicators (like heart rate variability, galvanic skin response) to create more comprehensive cognitive assessment frameworks. The study employed a single design task (intelligent walking cane design), and findings may vary across different types of creative challenges, complexity levels, or design domains. Future investigations should examine AIGC effects across diverse creative tasks, ranging from conceptual ideation to technical problem-solving, and across various design disciplines (e.g., industrial design, graphic design, architectural design) to establish broader applicability and identify task-specific optimization strategies.

## Data Availability

The original contributions presented in the study are included in the article/Supplementary material, further inquiries can be directed to the corresponding author.
